# Retinoblastoma: Molecular Evaluation of Tumor Samples, Aqueous Humor, and Peripheral Blood Using a Next-Generation Sequence Panel

**DOI:** 10.3390/ijms26083523

**Published:** 2025-04-09

**Authors:** Thais Biude Mendes, Indhira Dias Oliveira, Francine Tesser Gamba, Fernanda Teresa Lima, Bruna Fernanda Silva Cardoso Morales, Carla Renata Donato Macedo, Luiz Fernando Teixeira, Silvia Regina Caminada de Toledo

**Affiliations:** 1 Genetics Laboratory, Pediatric Oncology Institute (IOP/GRAACC), Federal University of Sao Paulo, Sao Paulo 04023-062, SP, Brazil; thais.biude.mendes@gmail.com (I.D.O.); francinegamba@graacc.org.br (F.T.G.); fernanda.teresa.lima@gmail.com (F.T.L.); 2 National Science and Technology Institute for Children’s Cancer Biology and Pediatric Oncology—INCT BioOncoPed, Porto Alegre 90035-003, RS, Brazil; 3 Department of Gynecology, Federal University of Sao Paulo, Sao Paulo 04024-002 SP, Brazil; 4 Institute of Pediatric Oncology (IOP/GRAACC), Sao Paulo 04023-062, SP, Brazil; brunacardoso@graacc.org.br (B.F.S.C.M.); carladonatomacedo@uol.com.br (C.R.D.M.); luizfteixeira@hotmail.com (L.F.T.); 5 Ophthalmology Department, Federal University of Sao Paulo, Sao Paulo 04023-062, SP, Brazil

**Keywords:** retinoblastoma, liquid biopsy, next-generation sequence panel, aqueous humor

## Abstract

Retinoblastoma was one of the first malignant tumors to be described as a genetic disease and its development occurs from the loss of function of the retinoblastoma gene (RB1). The difficulty in accessing the tumor during diagnosis highlights the need for non-invasive diagnostic methods. Studies have shown that liquid biopsy, obtained from any fluid material in the body, for example blood, contains free tumor cells and free and circulating DNA or RNA, making it a convenient tool for diagnosis and prognosis during cancer treatment without the need for invasive procedures. Taking advantage of these events, given this situation, we investigated molecular alterations in samples from retinoblastoma cases, using the NGS strategy as a powerful tool for characterization and aid in diagnosis and prognosis. Genomic data from 76 patients diagnosed with retinoblastoma, comprising 162 samples, tumor (TU), aqueous humor (AH), and peripheral blood (PB), were analyzed using the Oncomine Childhood Cancer Research Panel (OCCRA^®^). A total of 22 altered genes were detected, and 54 variants. Of the 76 cases, 29 included paired tumor (TU), aqueous humor (AH), and peripheral blood (PB) samples from the same patient. Alterations in the *RB1* gene were detected in 16 of these 29 cases, with concordant alterations identified across all three sample types in three patients. In 12 out of 29 patients, the same genetic alteration was found in both TU and AH. In conclusion, the OCCRA panel enabled the detection, in different samples, of molecular alterations in the *RB1* gene, as well as CNAs in the *MYCN*, *ABL2,* and *MDM4* genes. Limitations of AH were observed, primarily due to the small volume of material available and the consequently low concentration of cell-free DNA (cfDNA). However, as AH provides a viable alternative for analyzing tumors, inaccessible to traditional biopsy methods, liquid biopsy holds significant potential to improve diagnostic accuracy and guide treatment strategies in retinoblastoma cases.

## 1. Introduction

Retinoblastoma (RB) is a rare childhood intraocular cancer, unilateral or bilateral, manifested in children under 5 years old and encompassing 2–4% of childhood cancers [1,2]. This tumor typically develops due to the loss of function of the *RB1* gene, caused by single-nucleotide variants (SNVs), copy number variants (CNVs), loss of heterozygosity (LOH), or hypermethylation of the promoter. RB diagnosis is primarily clinical, relying on the visualization of the tumor mass, as performing a tumor biopsy using fine-needle aspiration (FNA) cytology is not feasible due to the risk of tumor dissemination beyond the eye [1,3,4,5,6].

The difficulty in accessing the tumor during diagnosis highlights the need for non-invasive diagnostic methods. Identifying biomarkers through non-invasive biopsies could represent a significant advancement in RB diagnosis. Studies have demonstrated that liquid biopsy, obtained from any fluid material in the body, for example blood, contains free tumor cells and cell-free and circulating DNA or RNA, making it a convenient tool for diagnosis and prognosis during cancer treatment without requiring invasive procedures [7].

Following the development of a safe protocol for removing aqueous humor (AH) from the eye minimizes intraocular pressure and avoids tumor seed reflux [8,9]. AH has emerged as an important source of tumor-derived genetic material. Subsequent analyses have demonstrated the presence of somatic mutations in the *RB1* gene in AH samples, consistent with findings from enucleated tumor tissue. These findings confirm the potential of AH as a liquid biopsy source for RB [10,11,12,13,14,15].

Advancements in next-generation sequencing (NGS) technologies have significantly enhanced cancer diagnosis and treatment strategies. Taking advantage of these events, given this situation, we investigated molecular alterations in different samples (tumor, aqueous humor, and from retinoblastoma 76 cases using the NGS strategy as a powerful tool for characterization and aid in diagnosis and prognosis as well the genomic concordance among the samples of 29 cases.

Genomic data from 76 patients diagnosed with retinoblastoma, comprising 162 samples, were analyzed. The Oncomine Childhood Cancer Research Panel (OCCRA^®^) evaluated the samples, which detected alterations in 200 genes identified as cancer drivers, a total of 22 altered genes, and 54 variants. Alterations in the *RB1* were observed in 51% of patients. A notable aspect of this study was the inclusion of paired samples. Of the 76 cases, 29 included paired tumor (TU), aqueous humor (AH), and peripheral blood (PB) samples from the same patient. Alterations in the *RB1* gene were detected in 16 of these 29 cases, with concordant alterations identified across all three sample types in three patients. In 12 out of 29 patients, the same genetic alteration was found in both TU and AH.

Alterations in nine other genes were observed, with *ABL2* and *MYCN* being the most frequently altered. The OCCRA panel successfully detected molecular alterations in the *RB1* gene, as well as copy number alterations (CNAs) in the *MYCN*, *ABL2*, and *MDM4* genes. These findings underscore the utility of AH as a viable alternative for analyzing tumors that are inaccessible to traditional biopsy methods. Liquid biopsy offers significant potential to improve diagnostic accuracy and guide treatment strategies in retinoblastoma cases.

## 2. Results

### 2.1. Clinical and Tumor Characteristics

In this study, we analyzed 76 patients diagnosed with retinoblastoma (RB), 43 (57%) patients were males and 33 (43%) females. Among these cases, 58 cases (77%) with unilateral disease, 17 cases (22%) were bilateral and 1 case (1%) was trilateral (bilateral retinoblastoma and pineoblastoma) (Table 1). The median age at diagnosis was 29 months (2.4 years), with unilateral retinoblastoma cases (2.7 years) showing a higher mean age compared to bilateral cases (1.7 years), resulting in a one-year difference (Table 1). Two cases were familial: the first case RB10, is a male, bilateral RB diagnosed at 3 years. The second case RB65, male, bilateral RB, diagnosed at 2 years, whose relatives, father and aunt, were diagnosed with unilateral RB at 11 months and 4 years, respectively (Table 1).

All patients have been classified based on the International Intraocular Retinoblastoma Classification (IIRC) at diagnosis. We observed 5% group A (5 eyes), 6% group B (6 eyes), 1% group C (1 eye), 5% group D (5 eyes) and 62% group E (58 eyes). When comparing unilateral and bilateral diseases, we observed that 79% of unilateral (46 cases) occurred in group E; and in bilateral, we found that 32% occurred in group E and 12% in groups A, B, and D (Table 1).

Of the 58 patients with unilateral disease, 48 (83%) had intraocular tumors and 10 (17%) had extraocular tumors. Of 18 patients with bilateral and trilateral tumors, 12 (67%) had intraocular and 6 (33%) had extraocular diagnosis. Primary enucleation was performed in 61 patients (80%), 91% (53 cases) unilateral, and 44% (8 cases) bilateral, who underwent enucleation before chemotherapy. Only 1 of 18 bilateral diseases underwent bilateral enucleation. Recurrence or metastasis was observed in 16% of patients (12 cases), and 8/76 patients died (Table 1). Of the eight patients died, six had recurrence to the central nervous system.

### 2.2. Identification of Genetic Alteration by Panel in Liquid Biopsy

**Figure 1:**
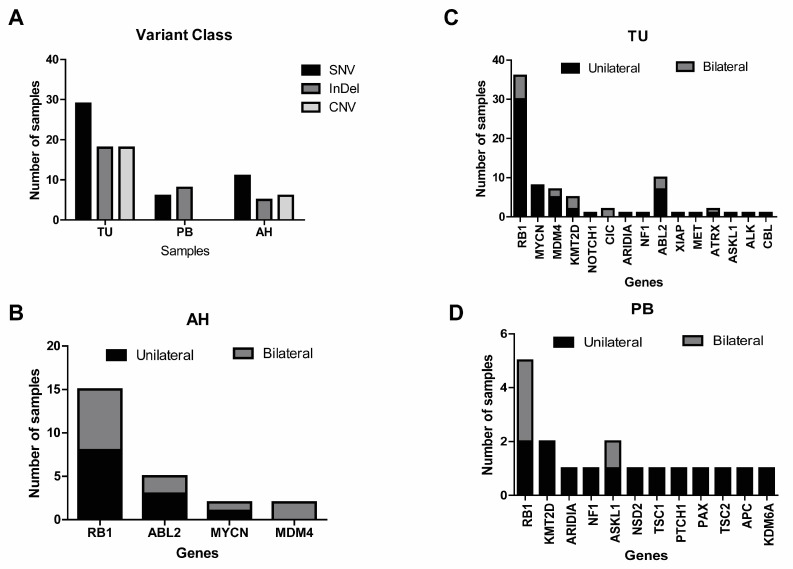
Genetic alterations identified in samples. Frequency of variant class (**A**) and genetic alterations identified in aqueous humor (**B**), tumor (**C**), and peripheral blood (**D**).

A total of 39 AH samples, derived from 38 out of 76 patients, were available for this study. The samples were collected from enucleated eyes. Cell-free DNA (cfDNA) was detected in all evaluated samples, with a mean concent1ration of 36.8 ng/µL (range 0.21–220 ng/µL). Genetic variants were investigated in all AH samples using the OCCRA^®^ panel, revealing genetic alterations in 17 of the 39 samples (44%), 11 samples with SNVs, 6 samples InDel and 6 samples CNVs being the most prevalent (Figure 1A). Among these samples, the most common genetic variants involved the *RB1* gene in 15/17 samples, 8 unilateral retinoblastoma samples and 7 bilateral retinoblastoma samples (Figure 1B). Specifically, 10 pathogenic somatic *RB1* variants and 4 *RB1* deletions were observed (Table 2). Additional alterations included copy number alteration (CNAs) involving the *ABL2*, *MYCN*, and *MDM4* genes, with the last gene observed exclusively in bilateral cases (Figure 1B). In cases RB50 and RB51, no *RB1* variant was detected, but CNAs in *ABL2* and *MYCN* genes were identified, respectively (Supplementary Figure S1).

### 2.3. Identification of Genetic Alterations in Tumor and Peripheral Blood

A total of 125 samples, comprising 69 tumors (TU) and 56 peripheral blood (PB) samples from 76 patients, were comprehensively evaluated using the OCCRA^®^ panel. Alterations were detected at an average rate of 0.7 per TU sample (range 0–3) and 0.3 per PB sample (range 0–2). The genomic landscape of RB patients can be observed in Supplementary Figure S1. No genetic alterations were detected in 61/125 samples (49%; 21 TU and 40 PB) using the OCCRA^®^ panel. In the remaining 64 samples (51%), alterations were identified in 22 (Figure 1A,C,D), with a total of 48 genetic variants detected, comprising 19 SNV, 18 insertions/deletions (InDels), and 4 CNAs. When analyzing all altered genes, it was observed that 9 out of 15 genes in tumor samples and 10 out of 12 genes in peripheral blood samples were found exclusively in patients with unilateral retinoblastoma (Figure 1C).

Alterations in the *RB1* gene were found in 32% of the samples (35 TU and 5 PB—Figure 1C), specifically, 16 SNV and 11 InDels (Table 3). The c.1333C>T and c.958>T variants were the most frequent in the TU samples (Table 3). Among the 35 tumors with *RB1* gene alteration, 21 contained only *RB1* variants, while 14 exhibited *RB1* alterations along with one or two additional alterations in other genes (Supplementary Figures S1 and S2A). Co-occurring alterations in *RB1* and seven genes were identified, with the most common being CNAs in *MYCN* and *MDM4* (Supplementary Figure S2B).

In 12 tumors and 11 peripheral blood samples, from 21 patients, no *RB1* alterations were detected, but other gene alterations were identified. The most common genes affected were *MYCN* and *ABL2* genes in TU samples and *ASKL1* and *KMT2D* in PB samples (Supplementary Table S1). In case RB66, where only a TU sample was available, three alterations were identified: one InDel in the gene *CBL*, and two CNAs in *ABL2* and *MDM4* (Supplementary Figure S1).

### 2.4. Genetic Analysis from Tumors, Aqueous Humor, and Peripheral Blood

**Figure 2:**
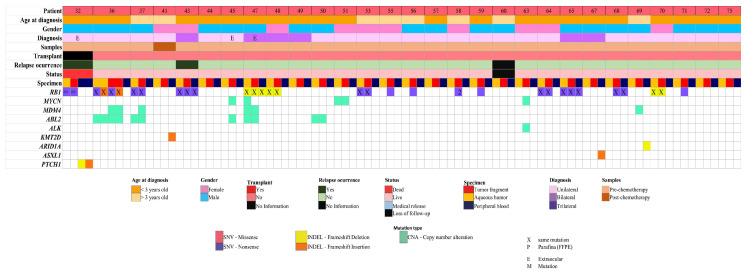
Genomic landscape of 29 RB cases paired samples (TU, AH, and PB).

A total of 29 cases (38%; 23 unilateral and 6 bilateral diseases) out of 76 cases had paired samples (TU, AH, and PB) (Figure 2). No genetic alterations were detected in six cases (21%). Mutation analysis identified 10 SNVs and 4 InDel in the *RB1* gene across 55% (16/29) cases (Table 4). Among the 16 cases, *RB1* gene alterations were observed in all samples from three patients (RB43, RB47, and RB65) (Table 4). In case RB47, with a diagnosis of bilateral disease and bilateral enucleation, AH from both eyes was evaluated, and just TU from the left eye. The evaluation revealed not only *RB1* gene alterations but also amplification of the *ABL2* and *MDM4* genes in the TU and AH samples. Additionally, amplification of the *MYCN* gene was observed exclusively in the AH sample from the right eye (Supplementary Table S2). In the familial case RB65, *RB1* gene alterations (specifically the c.1597G>T mutation) were detected consistently across all analyzed samples (TU, AH, and PB).

In 7 of the 16 cases with *RB1* gene alterations, changes were observed exclusively in TU and AH samples (Table 4). Additionally, two cases (RB36 and RB37) showed CNA in *ABL2* and *MDM4* genes, with the latter alteration found only in the tumor sample (Figure 2 and Supplementary Table S2). Patient RB32 exhibited a deletion (c.4324_4325delCG) and an insertion (c.921_922insC) in the *PTCH1* gene, which was identified exclusively in the PB sample (Supplementary Table S2).

In seven patients without *RB1* alterations, variations were identified in nine other genes, with the *ABL2* and *MYCN* being the most frequently altered (Supplementary Table S2). In patient RB69, alterations varied between TU and PB samples—a CNA in the *MDM3* gene was observed in the TU, whereas two InDels in the *ARID1A* gene were identified in the PB sample.

## 3. Discussion

Aqueous humor (AH) is an important source of tumor-derived genetic material, underscoring its potential use in liquid biopsies for retinoblastoma, particularly for detecting mutations in the *RB1* gene, as recently demonstrated [10,11,16,17,18]. In our study, genetic alterations in retinoblastoma samples were analyzed using a comprehensive next-generation sequencing (NGS) panel specific for pediatric neoplasms. A total of 164 samples (TU, PB, and AH) from a cohort of 76 patients were evaluated. Within this cohort, 60% of patients were diagnosed in group E retinoblastoma, the most advanced stage of retinoblastoma as classified by the IIRC [19].

As far as we know, this was the first study to employ genomic profiling using the OCCRA panel on different sample types (tumor, aqueous humor, and peripheral blood) from RB cases to investigate their molecular characteristics. The ion Torrent Oncomine Childhood Cancer Research Assay is a unique NGS-based tool designed for comprehensive genomic profiling of cancer affecting children and young adults. The assay is designed to provide researchers with sensitive and comprehensive sample amplification of relevant DNA mutations and fusion transcripts associated with childhood and young adult cancers in a single NGS run. The panel comprises 203 unique genes: 130 key DNA genes, 28 copy number variant targets, an expansive fusion panel of 90 driver genes with multiple partners, and 9 expression genes and controls. These cover the most relevant targets in the vast majority of all childhood and young adult oncology research samples [20]. For liquid biopsy, which involves low DNA concentrations, the method demonstrated the ability to detect genetic alteration in cell-free DNA (cfDNA) from AH, with high sensibility and coverage.

The findings revealed somatic genetic variants in 70% of TU samples, 29% of PB samples, and 44% of AH samples. A total of 22 altered genes were identified, with the most frequently detected variants being *RB1*, *ABL2*, *MYCN*, and *MDM4* genes. Genetic alterations in the *RB1* gene were identified in 33% of the samples. The most frequent genetic alterations, in our samples, were the pathogenic variants *RB1*: c.958C>T and c.1333C>T. The c958C>T mutation was detected in five cases of unilateral diseases. Interestingly, this mutation was found in both samples, TU and AH, of patient RB53. In the literature, this mutation has been reported in cases of unilaterally, being present in 5% of tumor samples analyzed [21]. It has also been described in familial RB cases as a mosaic mutation in 2–10% of the study’s cases [22,23]. The c.1333C>T mutation was detected in five cases, including four unilateral cases (four TU and one AH) and e one bilateral case (one AH). This mutation has already been described in four unilateral cases of patients from Singapore and four bilateral cases from India [22,24]. In the cases from India, three patients presented disease progression, and one required bilateral enucleation because he did not respond to treatment [24].

Previous research has demonstrated that somatic copy number alterations (SCNAs) may contribute to tumor progression in retinoblastoma cases [25]. CNAs in *ABL2*, *MDM3*, and *MYCN* genes were identified in 18 tumor samples. Among these, 4 out of 18 patients exhibited alterations in the *RB1* gene as well as CNAs in *MDM4* or *MYCN* genes. Two patients that had central nervous system recurrence and have died, have presented SNVs and InDELs in the *RB1* gene and CNVs in *ABL2* and *MDM4*, both.

The most recurrent SCNAs in RB include gains in 1q, 2p, 6p; losses in 13q e 16q; and *MYCN* gene amplifications [26,27]. The amplification of *MDM2* and *MDM4* genes may play an important role in retinoblastoma tumorigenesis, as these genes regulate p53 stability, as well as regulating the protein expression levels of the RB gene [28]. *ABL2* amplification has been reported in primary solid and hematologic tumors and in medulloblastoma, the expression of *ABL1* and *ABL2* has been associated with shorter survival rates [29].

Liquid biopsy can assist in the diagnosis and prognosis of patients with retinoblastoma, contributing to treatment planning based on the results. As previously described, findings such as *MYCN* gene amplification may indicate the importance of enucleation, as this gene has been reported to be associated with disease progression [10].

A notable aspect of this study is the evaluation of paired TU, AH, and PB samples from 29 out of 76 patients. Concordance of molecular findings across the three sample types from the same patient, obtained in the same day, was observed in 10% of the cases (3/29). In two cases (RB43 e RB65), the same SNV in the *RB1* gene was identified in all three sample types from each patient. In one third case, paired TU and AH samples from both eyes were analyzed. All five samples evaluated, from both eyes (TU and AH) and peripheral blood presented the same *RB1* mutation, while the TUs and AHs (from left and right eyes) exhibited concordant CNVs in *ABL2* and *MDM4*. Interestingly, a copy number gain of the *MYCN* gene was found exclusively in the right AH sample.

We found 28% of the TU and AH samples with molecular concordance. Specifically, six cases presented SNV or InDELs in the *RB1* gene in TU and AH, but not in PB. Additionally, two cases (RB36 and RB37) exhibited identical molecular alterations across the compared sample types.

In conclusion, the OCCRA panel enabled the detection, in different samples, of molecular alterations in the *RB1* gene, as well as CNAs in the *MYCN*, *ABL2*, and *MDM4* genes. Limitations of aqueous humor (AH) were observed, primarily due to the small volume of material available and the consequently low concentration of cell-free DNA (cfDNA). However, as AH provides a viable alternative for analyzing tumors inaccessible to traditional biopsy methods, liquid biopsy holds significant potential to improve diagnostic accuracy and guide treatment strategies in retinoblastoma cases.

## 4. Methods

### 4.1. Patients and Samples

A total of 162 samples (tumor (TU), aqueous humor (AH), and peripheral blood (PB), from 76 patients diagnosed with RB at Pediatric Oncology Institute-Grupo de Apoio ao Adolescente e à Criança com Câncer/Federal University of São Paulo (IOP-GRAACC/UNIFESP), between 2002 and 2022, were used in this study. TU and AH samples were collected after enucleated eye surgery. All samples belong to the Pediatric Oncology Institute Biobank IOP/GRAACC/UNIFESP (National Commission of Ethics in Research—CONEP B-053). This study was approved by the Institutional Research Committee at the Federal University of Sao Paulo (n° 0715P/2021).

### 4.2. DNA and RNA Extraction from Tumor and Peripheral Blood

Total DNA and RNA from 68 TU and 55 PB samples were extracted using AllPrep DNA/RNA Mini Kit (QIAGEN, Dusseldorf, Germany). DNA and RNA were quantified using the Nanodrop 2000 and Qubit 3 fluorometer (Life Technologies, Carlsbad, CA, USA). Quality thresholds for DNA and RNA were A260/2680 ratios of 1.6–18.8 and 1.8–2.0, respectively.

### 4.3. Cell-Free DNA/RNA Isolation from Aqueous Humor

Cell-free DNA/RNA was extracted from 39 AH samples. A volume of 100 µL AH was centrifuged at 2000× *g* for 10 min at 4 °C, supernatant was transferred to a new tube and centrifuged at 1400× *g* for 10 min. Then, the supernatant was processed by the QIAamp ccfDNA/RNA kit (QIAGEN, Dusseldorf, Germany), according to the manufacturer’s instructions. The samples were measured using the Quantus Fluorometer (PROMEGA, Madison, WI, USA).

### 4.4. cDNA Synthesis

Sequencing libraries were prepared through complementary DNA (cDNA) synthesis. To isolate RNA specifically from aqueous humor (AH), 10 µL of previously extracted nucleic acid was processed using a genomic DNA elimination protocol with the QuantiTect Reverse Transcription Kit, following the manufacturer’s instructions.

For cDNA synthesis, 20 ng of total RNA isolated from TU, AH, and PB samples was used. The synthesis was performed using the SuperScript IV VILO Master Mix (Thermo Fisher Scientific, Waltham, MA, USA) according to the manufacturer’s protocol.

### 4.5. Library Preparation and Next-Generation Sequencing (NGS) Run with the OCCRA^®^ Panel

A total of 20 ng DNA (TU, AH, and PB) and 20 ng cDNA (TU and PB), previously described, were used to prepare the sequencing libraries, of the Oncomine Childhood Cancer Research Assay (OCCRA) (Thermo Fisher Scientific, Waltham, MA, USA) according to the manufacturer’s protocol. Library construction was conducted in the Ion Chef System (Thermo Fisher Scientific).

Samples were sequenced and analyzed in the Ion S5 instrument, then Ion Reporter conducted the variant calling and fusion detection analysis, as previously described [30,31]. Sequencing libraries were prepared from 20 ng (15 μL) of DNA and 20 ng (13 μL) of cDNA from each tumor sample and performed on the Ion Chef TM System© (Thermo Fisher Scientific, Waltham, MA, USA), following the manufacturer’s protocol.

### 4.6. Oncomine Childhood Cancer Research Assay (OCCRA©) Sequencing Panel Analysis

At the end of the sequencing run, analysis of the raw data generated by large-scale sequencing was performed and the quality of each reading was evaluated. The readings obtained were aligned with the human reference genome hg19/GHCh37. The generated BAM files were analyzed using IGV (Integrative Genomics Viewer) software 5.20. The VCF (Variant Call Format) files were obtained and the interpretative analysis was performed using the software Ion Reporter© (Thermo Fisher Scientific, Waltham, MA, USA), through a specific platform designed for the OCCRA© panel. Genetic variants were classified according to type (SNVs, InDels, CNVs, fusions, deletions), functional effect (missense, nonsense, synonymous, and frameshift), and clinical significance (pathogenic, likely pathogenic, uncertain significance, likely benign, and benign). The variant classification was performed according to FATHMM (Functional Analysis through Hidden Markoy Model) prediction. In the CNVs analysis, only samples with a MAPD (Median of Absolute Values of all Pairwise Differences) ≤ 0.35 were considered. Variant characteristics were evaluated using COSMIC (Catalog of Somatic Mutations in Cancer), ClinVar (Clinically Relevant Variation), GeneCards (Human Gene Database), and Varsome (The Human Genomics Community) databases.

### 4.7. Data and Statistical Analysis

Data analysis was performed using GraphPad Prism version 7.0 (GraphPad Software, San Diego, CA, USA) for Windows. OS curves were generated by applying the Kaplan–Meier method with a 95% confidence interval (95% CI), and then compared by Log-rank test. Statistical significance was taken as *p* < 0.05 (5%).

## Figures and Tables

**Table 1:** Clinical demographics of 76 patients with retinoblastoma included in this study.

	Retinoblastoma			
	Unilateral	Bilateral	Trilateral	All
**Number of patients (%)**	58 (77%)	17 (22%)	1 (1%)	76 (100%)
**Number of eyes**	58	34	2	94
**Mean age diagnosis (Months)**	32	20	15	29
**Gender**				
Male	32 (55%)	10 (59%)	0	43 (57%)
Female	28 (48%)	7 (41%)	1 (100%)	33 (43%)
**Familial RB**	0	2 (11%)		2 (3%)
**Tumor**				
Intraocular	48 (83%)	11 (65%)	1 (100%)	60 (79%)
Extraocular	10 (17%)	6 (35%)	0	16 (21%)
**Primary treatment**				
Enucleation	53 (91%)	8 (47%)	0	61 (80%)
Therapy	5 (9%)	9 (53%)	1 (100%)	15 (20%)
**Recurrence**	4 (7%)	7 (41%)	1 (100%)	12 (16%)
**Mortality**	5 (9%)	3 (18%)	0	8 (10%)
**IIRC group**				
A	1 (2%)	4 (12%)	0	5 (5%)
B	2 (3%)	4 (12%)	0	6 (6%)
C	0	1 (3%)	0	1 (1%)
D	0	4 (12%)	1 (50%)	5 (5%)
E	46 (79%)	11 (32%)	1 (50%)	58 (62%)
Without group	9 (15%)	10 (29%)	0	19 (21%)

**Table 2:** Types of genetic variants in *RB1* gene in AH of 15 cases with retinoblastoma.

Patient ID	Laterality	Initial Treatment	Gene	Chromosome	Coding	Aminoacid Change	Variant Classification	Gene Classification	Variant Class	Variant Effect	Clinical Significance
RB32	UL	PE	*RB1*	13q14.2	c.160G>T	p.Glu54Ter	Deletion	Lost	SNV	Nonsense	Pathogenic
				13q14.2	c.1735C>T	p.Arg579Ter	Deletion	Lost	SNV	Nonsense	Pathogenic
RB36	UL	PE	*RB1*	13q14.2	c.1072C>T	p.Arg358Ter	Deletion	Lost	SNV	Nonsense	Pathogenic
				13q14.2	c.1341_1342insA	p.Leu448fs	Deletion	Lost	INDEL	Frameshift Insertion	Patogenic
RB37	UL	PE	*RB1*	13q14.2	c.1333C>T	p.Arg445Ter	Deletion	Lost	SNV	Nonsense	Patogenic
RB40	BL	IVC	*RB1*	13q14.2	c.1333C>T	p.Arg445Ter	Deletion	Lost	SNV	Nonsense	Patogenic
RB43	BL	IVC	*RB1*	13q14.2	c.361C>T	p.Gln121Ter	Deletion	Lost	SNV	Nonsense	
RB46	BL	IVC	*RB1*	13q14.2	c.1072C>T	p.Arg358Ter	Deletion	Lost	SNV	Nonsense	Patogenic
RB47 OD	BL	PE	*RB1*	13q14.2	c.933delT	p.Pro312fs	Deletion	Lost	INDEL	Frameshift Deletion	Patogenic
RB47 OE	BL	PE	*RB1*	13q14.2	c.933delT	p.Pro312fs	Deletion	Lost	INDEL	Frameshift Deletion	Patogenic
RB48	BL	PE	*RB1*	13q14.2	c.2486delC	p.Ser829Ter	Deletion	Lost	INDEL	nonsense	Patogenic
RB53	UL	PE	*RB1*	13q14.2	c.958C>T	p.Arg320Ter	Deletion	Lost	SNV	Nonsense	Patogenic
RB54	UL	PE	*RB1*	13q14.2	c.1318G>T	p.Glu440Ter	Truncating mutation	Lost	SNV	Nonsense	Patogenic
RB64	UL	PE	*RB1*	13q14.2	c.1363C>T	p.Arg455Ter	Truncating mutation	Lost	SNV	Nonsense	Patogenic
RB65	BL	PE	*RB1*	13q14.2	c.1597G>T	p.Glu533Ter	Deletion	Lost	SNV	Nonsense	Patogenic
RB70	UL	PE	*RB1*	13q14.2	c.2478delT	p.Pro827GlnfsTer6	Truncating mutation	Lost	INDEL	Frameshift Deletion	Patogenic
RB73	UL	PE	*RB1*	13q14.2	c.1654C>T	p.Arg552Ter	Truncating mutation	Lost	SNV	Nonsense	Patogenic

IVC = systemic intravenous chemotherapy. PE = primary enucleation.

**Table 3:** Genomic alterations identified in the *RB1* gene in the samples TU and PB.

Coding	Aminoacid Change	Classificação Variante	Classificação Gene	Variant Class	Variant Effect	Clinical Significance	TU	PB
c.1654C>T	p.Arg552Ter	Deletion	Lost	SNV	Nonsense	Pathogenic	1	0
c.1333C>T	p.Arg455Ter	Deletion	Lost	SNV	Nonsense	Pathogenic	4	0
c.1363C>T	p.Arg455Ter	Deletion	Lost	SNV	Nonsense	Pathogenic	3	0
c.958C>T	p.Arg320Ter	Deletion	Lost	SNV	Nonsense	Pathogenic	5	0
c.1666C>T	p.Arg556Ter	Deletion	Lost	SNV	Nonsense	Pathogenic	2	1
c.1072C>T	p.Arg358Ter	Deletion	Lost	SNV	Nonsense	Pathogenic	3	0
c.1060C>T	p.Gln354Ter	Deletion	Lost	SNV	Nonsense	Pathogenic	1	0
c.844G>T	p.Glu282Ter	Deletion	Lost	SNV	Nonsense	Pathogenic	1	0
c.1735C>T	p.Arg579Ter	Deletion	Lost	SNV	Nonsense	Pathogenic	1	0
c.160G>T	p.Glu54Ter	Deletion	Lost	SNV	Nonsense	Pathogenic	1	0
c.526C>T	p.Gln176Ter	Deletion	Lost	SNV	Nonsense	Pathogenic	1	0
c.361C>T	p.Gln121Ter	Truncating mutation	Lost	SNV	Nonsense	Pathogenic	2	1
c.1318G>T	p.Glu440Ter	Truncating mutation	Lost	SNV	Nonsense	Pathogenic	1	0
c.1597G>T	p.Glu533Ter	Truncating mutation	Lost	SNV	Nonsense	Pathogenic	1	1
c.1330C>T	p.Gln444Ter	Truncating mutation	Lost	SNV	Nonsense	Pathogenic	1	1
c.2308C>T	p.Gln770Ter	Truncating mutation	Lost	SNV	Nonsense	Pathogenic	1	0
c.2532_2541delGTTCCAGAAA	p.Phe845Ter	Deletion	Lost	InDel	Nonsense	Pathogenic	1	0
c.1942delT	p.Ser648fs	Deletion	Lost	InDel	Frameshift deletion	Pathogenic	1	0
c.795delA	p.Lys265fs	Deletion	Lost	InDel	Frameshift deletion	Pathogenic	1	0
c.733_739delCCCATTA	p.Pro245fs	Deletion	Lost	InDel	Frameshift deletion	Pathogenic	1	0
c.660_661delAT	p.Val222fs	Deletion	Lost	InDel	Frameshift deletion	Pathogenic	1	0
c.1827delT	p.Val610Ter	Deletion	Lost	InDel	Nonsense	Pathogenic	1	0
c.1341_1342insA	p.Leu448fs	Deletion	Lost	InDel	Frameshift insertion	Pathogenic	1	0
c.933delT	p.Pro312fs	Deletion	Lost	InDel	Frameshift deletion	Pathogenic	1	1
c.2486delC	p.Ser829Ter	Deletion	Lost	InDel	Nonsense	Pathogenic	1	0
c.613_614insGAAG	p.Val205fs	Deletion	Lost	InDel	Frameshift insertion	Pathogenic	1	0
c.2478delT	p.Pro827GlnfsTer6	Truncating mutation	Lost	InDel	Frameshift deletion	Pathogenic	1	0

**Table 4:** Types of genetic variants in the *RB1* gene in 16 cases with samples paired (TU, AH, and PB).

Patient ID	Gender	Laterality	Sample	Nucleotide Change	Amino acid Change	Variant Classification	Gene Classification	Variant Class	Consequences
RB 32	M	UL	TU/AH	c.160G>T	p.Glu54Ter	Deletion	Lost	SNV	Nonsense
				c.1735C>T	p.Arg579Ter	Deletion	Lost	SNV	Nonsense
RB 36	M	UL	TU/AH	c.1072C>T	p.Arg358Ter	Deletion	Lost	SNV	Nonsense
				c.1341_1342insA	p.Leu448fs	Deletion	Lost	InDel	Frameshift insertion
RB 37	M	UL	TU/AH	c.1333C>T	p.Arg445Ter	Deletion	Lost	SNV	Nonsense
RB 43	F	BL	TU/AH/PB	c.361C>T	p.Gln121Ter	Truncating mutation	Lost	SNV	Nonsense
RB 47	M	BL	TU/AH/PB	c.933delT	p.Pro312fs	Deletion	Lost	InDel	Frameshift deletion
RB 48	F	BL	TU/AH	c.2486delC	p.Ser829Ter	Deletion	Lost	InDel	Nonsense
RB 53	F	UL	TU/AH	c.958C>T	p.Arg320Ter	Truncating mutation	Lost	SNV	Nonsense
RB 55	F	UL	TU	c.361C>T	p.Gln121Ter	Truncating mutation	Lost	SNV	Nonsense
RB 56	M	UL	TU	c.958C>T	p.Arg320Ter	Truncating mutation	Lost	SNV	Nonsense
RB 58	F	UL	TU	c.958C>T	p.Arg320Ter	Truncating mutation	Lost	SNV	Nonsense
				c.1072C>T	p.Arg358Ter	Deletion	Lost	SNV	Nonsense
RB 59	M	UL	TU	c.958C>T	p.Arg320Ter	Truncating mutation	Lost	SNV	Nonsense
RB 64	F	UL	TU/PB	c.1363C>T	p.Arg455Ter	Truncating mutation	Lost	SNV	Nonsense
RB 65	M	BL	TU/AH/PB	c.1597G>T	p.Glu533Ter	Truncating mutation	Lost	SNV	Nonsense
RB 68	M	UL	TU/PB	c.1330C>T	p.Gln444Ter	Truncating mutation	Lost	SNV	Nonsense
RB 70	F	UL	TU/AH	c.2478delT	p.Pro827GlnfsTer6	Truncating mutation	Lost	InDel	Frameshift deletion
RB 71	M	UL	TU	c.2308C>T	p.Gln770Ter	Truncating mutation	Lost	SNV	Nonsense

## Data Availability

The data presented in this study are available within the article.

## Supplementary Materials

The following supporting information can be downloaded at: https://www.mdpi.com/article/10.3390/ijms26083523/s1.
