# Supplementary material for: Retinoblastoma: Molecular Evaluation of Tumor Samples, Aqueous Humor, and Peripheral Blood Using a Next-Generation Sequence Panel
Source: Int J Mol Sci. 2025 Apr 9;26(8):3523. doi: 10.3390/ijms26083523 (PMC12027083; doi:10.3390/ijms26083523)
Supplement: Supplementary file 1 [file ijms-26-03523-s001.zip › Table Supplemental.pdf]

SUPPLEMENTAL MATERIAL - TABLES

TABLE S1: Genomic alterations identified in the samples TU and PB.

Gene	Chromosome	Coding	Aminoacid change	Variant Classification	Gene Classification	Variant class	Variant effect	Clinical significance	TU	PB
CIC	19q13.2	c.4533_4534delCCinsTT, c.4533C>T	p.Arg1512Cys, p.(=)	Hotspot	Gain	SNV, MNV	Synonymous, Missense, Synonymous	Benign	1	0
ATRX	Xq21.1	c.6391C>T	p.Arg2131Ter	Deletion	Lost	SNV	Nonsense	Patogenic	1	0
NSD2	4p16.3	c.4023C>T	***	**	**	SNV	Synonymous	Benign	0	1
ASXL1	20q11.21	c.3306G>T	p.Glu1102Asp	Hotspot	Lost	SNV	Missense	***	0	1
MET	7q31.2	c.2962C>T	p.Arg988Cys	Hotspot	Gain	SNV	Missense		1	0
PAX5	9p13.2	c.77T>G	p.Val26Gly	Hotspot	Gain	SNV	Missense	VOUS	0	1
ASXL1	20q11.21	c.1953_1954insA	p.Gly652ArgfsTer6	Truncating mutation	Lost	Indel	Frameshift Insertion	***	0	1
KMT2D	12q13.12	c.6554_6555insC	p.Glu2186fs	Deletion	Lost	Indel	Frameshift Insertion	Patogenic Probably	0	2
ARID1A	1p36.11	c.3428_3429delGG	p.Gly1143AlafsTer49	Truncating mutation	Lost	Indel	Frameshift deletion	Patogenic Probably	0	1
ARID1A	1p36.11	c.3429delG	p.Gln1145ArgfsTer16	Truncating mutation	Lost	Indel	Frameshift deletion	***	0	1
NF1	17q11.2	c.6462_6463insA	p.Glu2155fs	Deletion	Lost	Indel	Frameshift Insertion	Patogenic	0	1
NSD2	4p16.3	c.4028delC	p.Pro1343fs	Deletion	Lost	Indel	Frameshift deletion	Patogenic	0	1
TSC1	9q34.13	c.2921_2922insA	p.Leu975fs	Deletion	Lost	Indel	Frameshift Insertion	Patogenic	0	1
PTCH1	9q22.32	c.4324_4325delCG	p.Arg1442fs	Deletion	Lost	Indel	Frameshift deletion	Patogenic Probably	0	1
PTCH1	9q22.32	c.921_922insC	p.Ala308fs	Deletion	Lost	Indel	Frameshift Insertion	Patogenic	0	1

NTHL1 / TSC2	16p13.3	c.-867A>TA, c.102_103insA	p.?, p.Gln35fs	Deletion	Lost	Indel	Unknown, Frameshift Insertion	***	0	1
CBL	11q23.3	c.1380_1382delTGA	p.Asp460del	Hotspot	Gain	Indel	Nonframeshift Deletion	VOUS	1	0
ABL2	1q25.2	***	***	Amplification	Gain	CNV	***	***	4	0
MDM4	1q32.1	***	***	Amplification	Gain	CNV	***	***	1	0
MYCN	2p24.3	***	***	Amplification	Gain	CNV	***	***	3	0
ALK	2p23.2-p23.1	***	***	Amplification	Gain	CNV	***	***	1	0

VOUS - unknown clinical significance

TABLE S2: Genomic alterations identified in the paired (TU, AH, and PB).

Patient ID	Laterality	Sample	Gene	Chromosome	Coding	Aminoacid change	Variant Classification	Gene Classification	Variant class	Variant effect	Clinical significance
RB 32	UL	PB	*PTCH1*	9q22.32	c.4324_4325delCG	p.Arg1442fs	Deletion	Lost	Indel	Frameshift Deletion	Patogenic Probably
				9q22.32	c.921_922insC	p.Ala308fs	Deletion	Lost	Indel	Frameshift Insertion	Patogenic
RB 36	UL	FG/AH	*ABL2*	1q25.2	***	***	Amplification	Gain	CNV	***	***
		FG	*MDM4*	1q25.2	***	***	Amplification	Gain	CNV	***	***
RB 37	UL	FG/AH	*ABL2*	1q25.2	***	***	Amplification	Gain	CNV	***	***
		FG	*MDM4*	1q25.2	***	***	Amplification	Gain	CNV	***	***
RB 41	UL	PB	*KMT2D*	12q13.12	c.6554_6555insC	p.Glu2186fs	Deletion	Lost	Indel	Frameshift Insertion	Patogenic Probably

RB45	UL	FG	*ABL2*	1q25.2	***	***	Amplification	Gain	CNV	***	***
			MYCN	2p24.3	***	***	Amplification	Gain	CNV	***	***
RB47	BL	FG/AH	*ABL2*	1q25.2	***	***	Amplification	Gain	CNV	***	***
		FG/AH	*MDM4*	1q32.1	***	***	Amplification	Gain	CNV	***	***
		AH	*MYCN*	2p24.3	***	***	Amplification	Gain	CNV	***	***
RB50	UL	FG/AH	*ABL2*	1q25.2	***	***	Amplification	Gain	CNV	***	***
RB51	UL	FG/AH	*MYCN*	2p24.3	***	***	Amplification	Gain	CNV	***	***
RB63	UL	FG	*MYCN*	2p24.3	***	***	Amplification	Gain	CNV	***	***
			ALK	2p23.2-p23.1	***	***	Amplification	Gain	CNV	***	***
RB67	BL	PB	*ASXL1*	20q11.21	c.1953_1954insA	p.Gly652ArgfsTer6	Truncating mutation	Lost	Indel	Frameshift Insertion	***
RB69	UL	FG	*MDM4*	1q32.1	***	***	Amplification	Gain	CNV	***	***
		PB	*ARID1A*	1p36.11	c.3428_3429delGG	p.Gly1143AlafsTer49	Truncating mutation	Lost	Indel	Frameshift Deletion	***
					c.3429delG	p.Gln1145ArgfsTer16	Truncating mutation	Lost	Indel	Frameshift Deletion	***
